# Ohio

**Published:** 1896-09

**Authors:** 


*Ohio.* Diphtheria is reported among the dogs at Cleveland, where it said to be causing serious loss among the kennels of valuable animals kept in that city.

